# Clinical applications of large language models in knee osteoarthritis: a systematic review

**DOI:** 10.3389/fmed.2025.1670824

**Published:** 2025-11-19

**Authors:** Zebing Ma, Yibing Liu, Ziyan Zhang, Rui Chen, Huayu Fan, Xiangyang Cao, Lili Ni

**Affiliations:** 1 Hunan University of Chinese Medicine, Changsha, Hunan, China; 2 Central South University, Changsha, Hunan, China; 3 Luoyang Orthopedic Hospital of Henan Province (Orthopedic Hospital of Henan Province), Zhengzhou, China; 4 Institute of Intelligent Medical and Bioengineering Henan Academy of Traditional Chinese Medicine Sciences, Zhengzhou, China; 5 Henan Province Artificial Intelligence Engineering Research Center for Bone Injury Rehabilitation, Zhengzhou, China; 6 Henan University of Chinese Medicine, Zhengzhou, Henan, China; 7 The Second Affiliated Hospital of Hunan University of Chinese Medicine, Changsha, Hunan, China

**Keywords:** knee osteoarthritis, large language models, artificial intelligence, clinical decision support, systematic review, ChatGPT

## Abstract

**Background and aims:**

Knee osteoarthritis (KOA) is a common chronic degenerative disease that significantly impacts patients’ quality of life. With the rapid advancement of artificial intelligence, large language models (LLMs) have demonstrated potential in supporting medical information extraction, clinical decision-making, and patient education through their natural language processing capabilities. However, the current landscape of LLM applications in the KOA domain, along with their methodological quality, has yet to be systematically reviewed. Therefore, this systematic review aims to comprehensively summarize existing clinical studies on LLMs in KOA, evaluate their performance and methodological rigor, and identify current challenges and future research directions.

**Methods:**

Following the PRISMA guidelines, a systematic search was conducted in PubMed, Cochrane Library, Embase databases and Web of science for literature published up to June 2025. The protocol was preregistered on the OSF platform. Studies were screened using standardized inclusion and exclusion criteria. Key study characteristics and performance evaluation metrics were extracted. Methodological quality was assessed using tools such as Cochrane RoB, STROBE, STARD, and DISCERN. Additionally, the CLEAR-LLM and CliMA-10 frameworks were applied to provide complementary evaluations of quality and performance.

**Results:**

A total of 16 studies were included, covering various LLMs such as ChatGPT, Gemini, and Claude. Application scenarios encompassed text generation, imaging diagnostics, and patient education. Most studies were observational in nature, and overall methodological quality ranged from moderate to high. Based on CliMA-10 scores, LLMs exhibited upper-moderate performance in KOA-related tasks. The ChatGPT-4 series consistently outperformed other models, especially in structured output generation, interpretation of clinical terminology, and content accuracy. Key limitations included insufficient sample representativeness, inconsistent control over hallucinated content, and the lack of standardized evaluation tools.

**Conclusion:**

Large language models show notable potential in the KOA field, but their clinical application is still exploratory and limited by issues such as sample bias and methodological heterogeneity. Model performance varies across tasks, underscoring the need for improved prompt design and standardized evaluation frameworks. With real-world data and ethical oversight, LLMs may contribute more significantly to personalized KOA management.

**Systematic review registration:**

https://osf.io/jy4kz, identifier 10.17605/OSF.IO/479R8.

## 1 Introduction

Knee osteoarthritis (KOA) is a prevalent and progressively exacerbating degenerative joint disorder, affecting over 250 million individuals worldwide. Among populations over the age of 40, the prevalence rate reaches approximately 22% (1). KOA is primarily characterized by the gradual degeneration of articular cartilage, osteophytic growth at joint margins, and inflammatory responses in periarticular soft tissues. These pathological changes result in chronic pain, reduced mobility, and significant impairment in quality of life (2). The diagnosis and treatment of KOA rely on a comprehensive array of information sources, including imaging data, clinical symptoms, medical history, and subjective patient feedback. However, traditional clinical workflows often suffer from high subjectivity, suboptimal decision-making efficiency, and challenges in data integration, highlighting the urgent need for more intelligent and streamlined decision-support tools to enhance diagnostic accuracy and therapeutic outcomes.

Recent advancements in artificial intelligence (AI), particularly the emergence of large language models (LLMs) such as ChatGPT, Gemini, and DeepSeek, have introduced transformative possibilities within the medical domain (3). Leveraging natural language processing, LLMs are capable of extracting clinical insights from unstructured medical texts, supporting personalized treatment planning, predicting disease prognosis, facilitating physician–patient communication, and contributing to public health education. Within the field of KOA, the potential of LLMs has begun to attract scholarly attention. Preliminary studies have explored their application in contexts such as medical record interpretation and automated radiology report generation. Nonetheless, a systematic evaluation of their clinical benefits, safety concerns, and limitations is lacking. Therefore, this study aims to comprehensively review current research on the application of LLMs in KOA, identify existing challenges and knowledge gaps, and propose future directions to guide the clinical translation of these technologies.

## 2 Methods

This study was prospectively registered on the Open Science Framework (associated project)^1^ and conducted in accordance with the PRISMA guidelines to ensure methodological rigor and transparency.

### 2.1 Literature search strategy

Two independent researchers (ZM and YL) conducted comprehensive literature searches to enhance both the sensitivity and specificity of the retrieval process. Searches were performed in PubMed, the Cochrane Library, Web of science and Embase, with language restricted to English and covering the period from database inception to June 2025. Search strategies were developed around the concepts of LLMs and KOA, using Boolean operators (OR and AND) to construct logical query expressions (Supplementary Data). All search procedures followed a predefined, standardized protocol and were cross-validated by a third researcher (ZZ) for consistency.

### 2.2 Inclusion and exclusion criteria

Inclusion criteria were as follows: (1) studies involving the real-world application of at least one LLM in the context of KOA; (2) articles classified as original research; (3) articles published in English.

Exclusion criteria included: (1) studies unrelated to KOA, or those focusing exclusively on other AI approaches (e.g., traditional machine learning) without incorporating LLMs; (2) non-original articles such as reviews, meta-analyses, conference abstracts, or case reports; (3) publications not written in English.

### 2.3 Study selection

Two researchers (ZM and YL) independently imported the retrieved records into EndNote (version 21) for reference management and screened studies based on the predefined inclusion and exclusion criteria. Discrepancies were resolved through discussion; if consensus could not be reached, a third researcher (ZZ) was consulted to make the final decision.

### 2.4 Quality and performance assessment

For all included studies, two reviewers (ZM and YL) independently conducted the quality assessment. An initial classification was performed based on the titles and abstracts, followed by the selection of appropriate appraisal tools according to the study design upon full-text review. Specifically, randomized controlled trials (RCTs) were assessed using the Cochrane risk of bias tool (4); diagnostic accuracy studies were assessed using the standards for reporting diagnostic accuracy studies (STARD) checklist (5); observational studies were assessed using the strengthening the reporting of observational studies in epidemiology (STROBE) guidelines (6); and educational or informational resources were assessed using the DISCERN instrument (7). In addition, guided by AI-specific reporting standards such as CONSORT-AI (8), we developed a checklist for evaluating the methodological quality of LLM-based research (CLEAR-LLM), specifically designed to address the unique characteristics of LLMs research. This secondary evaluation tool encompasses 12 dimensions, including model description, prompt design, human evaluation, output quantification, among others (Supplementary Data).

Given the absence of a standardized framework for assessing the output quality of LLMs in clinical applications (9), we synthesized components from existing instruments to construct the clinical language model assessment (CliMA-10). This tool comprises seven core domains: medical factual accuracy, contextual coherence, explainability of terminology, clinical applicability, hallucination mitigation, safety and ethics, and structured output capability. Additionally, it includes three supplementary adaptive domains. Each domain is rated on a 5-point Likert scale. The overall score is calculated as a weighted average, with core domains accounting for 60% and adaptive domains accounting for 40%. Within each category, equal weighting is applied: each core domain carries a weight of 60%/7, and each adaptive domain 40%/3 (Supplementary Data). All assessments were performed independently by two researchers; discrepancies were resolved through adjudication by a third researcher (ZZ).

### 2.5 Data extraction and synthesis

To ensure objectivity in data processing, extraction and synthesis were performed by two independent reviewers (RC and HF) who were not involved in the initial literature screening, in order to maintain methodological blinding. In cases of disagreement, a third reviewer (XC) intervened to facilitate consensus and make the final determination. All data were analyzed using Microsoft Excel (version 2019) and visualized via Adobe Illustrator (version 2022).

## 3 Results

### 3.1 Characteristics of included studies

**FIGURE 1:**
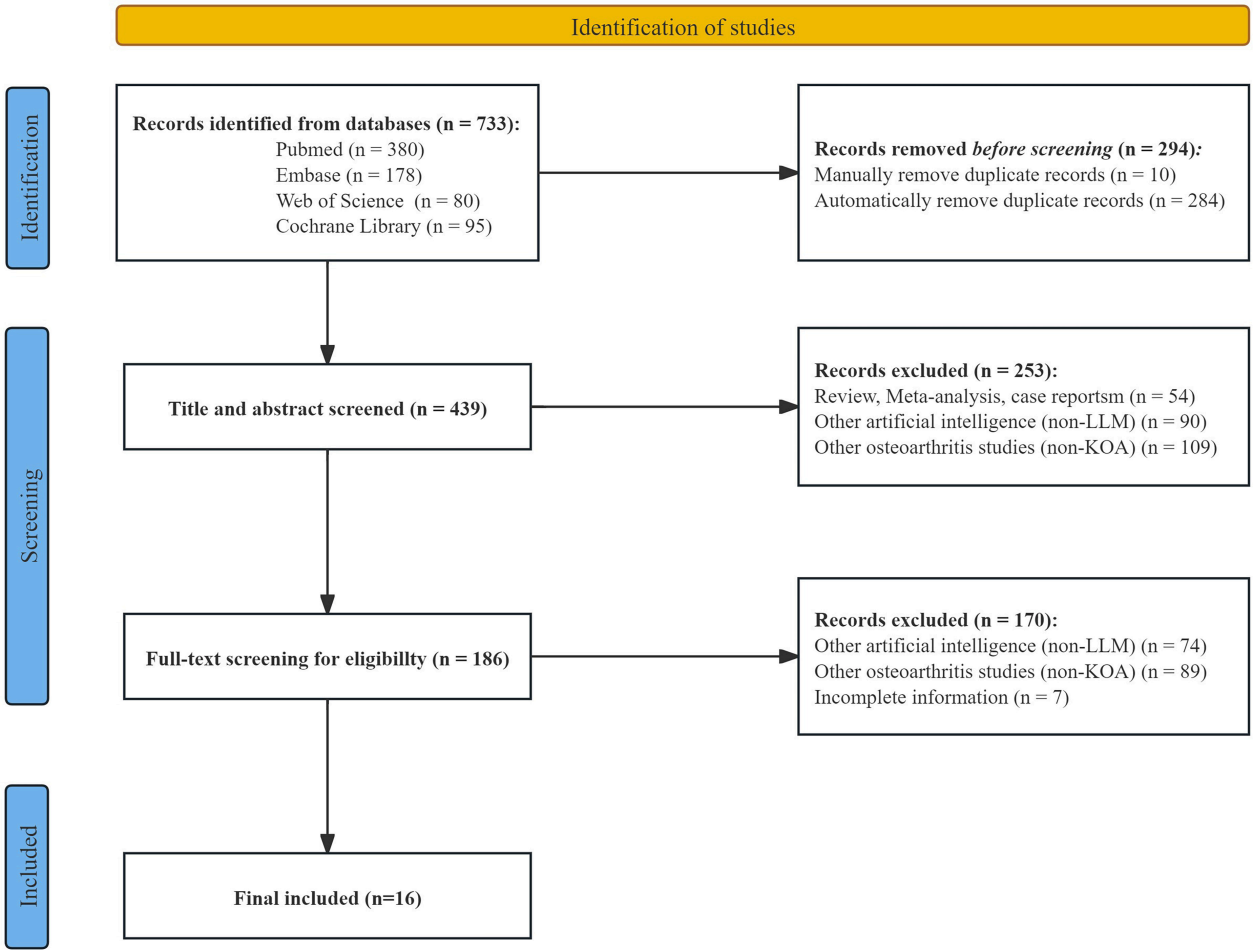
Flowchart of the study selection process.

From 2015 to 2025, research involving LLMs in the field of KOA has exhibited a steadily increasing trend. A total of 733 studies were initially retrieved. Following deduplication performed with EndNote (version 21.0), 439 records were retained. Title and abstract screening reduced the pool to 186 studies, and after full-text review, 16 studies were ultimately selected for systematic evaluation (Figure 1). Among the included studies, ChatGPT (e.g., ChatGPT-3.5, ChatGPT-4, ChatGPT-4o) were the most frequently employed LLMs, along with other models such as Gemini and Claude. These LLMs were applied across diverse KOA-related scenarios, including imaging-based diagnosis, clinical decision support, and patient education. Specific tasks included text generation, medical question answering, and generating personalized treatment recommendations. In terms of evaluation metrics, most studies adopted a comprehensive, multidimensional assessment framework, integrating both subjective and objective indicators.

Subjective metrics included answer quality, linguistic fluency, readability, and empathy, which were often assessed using Likert scales or content quality evaluation tools such as DISCERN. Objective metrics varied depending on the task and included diagnostic accuracy, sensitivity, area under the curve, and information extraction performance indicators such as precision, recall, and F1 score. The types of study samples were diverse, encompassing real or standardized patient cases, radiological data (MRI or X-ray), and clinical queries. Detailed characteristics are summarized in Table 1.

**TABLE 1:** Summary of applications and features of current LLMs in KOA.

Application scenario	Specific task	Model	Evaluation metrics	Type	Sample	References
Patient education	Improve KOA patients’ understanding of health knowledge	ChatGPT (version unspecified)	Patient knowledge score (visual analogue scale) GPT response quality score Number of patient-raised questions	Subjective	60 KOA patients	Yang et al. (23)
Imaging-assisted diagnosis	Extract structured scores from free-text MRI reports and predict KOA severity	GPT-4o GPT-4o mini (non-fine-tuned)	Structured report performance Accuracy of KOA severity prediction Subjective evaluation by orthopedic surgeons	Objective and subjective	160 KOA MRI reports	Xie et al. (21)
Personalized management	Generate personalized KOA self-management advice and compare with physician recommendations	ChatGPT-4	Quality evaluation, Likert 5 scare Blinded identification accuracy	Subjective	36 KOA clinical records	Du et al. (26)
Imaging-assisted diagnosis	Interpret K-L grade on knee X-rays and compare with physician diagnosis	ChatGPT-4o	Diagnostic performance Classifier metrics Model discriminative ability	Objective	200 knee X-rays	Temel et al. (14)
Imaging-assisted diagnosis	Automatically detect KOA and K-L grade levels from X-ray images	ChatGPT-4o	Detection accuracy Grading performance Performance variation by age and sex. Error analysis	Objective	117 knee X-rays	Zhu et al. (15)
Clinical decision support	Evaluate LLM’s ability in medical evidence summarization and structured expression	ChatGPT-4o GPT-4 Turbo GPT-4 mini GPT-3.5 Turbo Gemini 1.5 Gemini 1.0 Liama-3.1 Gemma 2 Mistral-Nemo	Medical accuracy, Likert 5 scare Usability, Likert 5 scare Risk metrics	Subjective	50 generated responses	Pagano at al. (13)
Patient education	Answer standard medical questions using lay-friendly language	ChatGPT-4 ChatGPT-4 (the knee guide)	Readability Information quality	Objective and subjective	50 generated responses	Fahy et al. (20)
Guideline interpretation	Assess LLM’s ability in summarizing medical content and answering guideline-based questions	ChatGPT-4	Response quality, Likert 3–5 scale Guideline alignment rate Case response quality, 4-level rating	Objective and subjective	108 guideline entries + 50 case entries	Li et al. (29)
Personalized therapy	Provide exercise guidance Q&A and explanation of individual training plans	ChatGPT-4	Compliance and safety Subjective experience, Likert 5 scale	Subjective	72 patients	You et al. (46)
Patient education	Automatically generate science-based Q&A from patient-submitted questions	ChatGPT-3.5, ChatGPT-4.0, Perplexity AI	Answer quality evaluation, Likert 5 scale Content structure and references Risk of misinformation and bias	Subjective	60 generated responses	Cao et al. (16)
Clinical decision support	Simulate preoperative consultations and assess surgical decisions	ChatGPT-3.5 ChatGPT-4 Microsoft Bing Gemini	Clinical decision concordance Model self-confidence, Likert 5 scale Quality and risk of answers	Objective and subjective	32 standardized virtual patients	Musbahi et al. (47)
Patient education	Generate patient-facing information on PRP treatment for KOA	ChatGPT-3.5, ChatGPT-4	Information quality Readability Consistency and reliability	Objective and subjective	15 generated responses	Fahy et al. (31)
Patient Education	Compare LLM generated content with authoritative guideline content	Bard ChatGPT (version unspecified)	Information quality Language quality, Likert scale Consistency	Subjective	48 generated responses	Yang et al. (25)
Clinical decision support	Grade diagnosis and provide treatment suggestions for standardized KOA cases	ChatGPT-4	Diagnostic and grading accuracy Treatment appropriateness Error bias detection	Objective and subjective	9 standardized KOA cases	Pagano et al. (24)
Self-diagnosis support	Diagnose based on patient symptom descriptions	ChatGPT-3.5	Diagnostic accuracy Structured output Error identification	Objective and subjective	1 KOA case (case 6)	Kuroiwa et al. (22)
Medical education	Assess LLM potential in assisting clinician learning and basic querying	ChatGPT-4o Gemini	Medical content quality, Likert 5 scale Structured expression, Likert 5 scale	Subjective	560 data points	Gürses et al. (30)

KOA, knee osteoarthritis; K-L, Kellgren-Lawrence; LLM, large language model; Q&A, question and answer; PRP, platelet-rich plasma.

### 3.2 Methodological quality assessment

The methodological evaluation was conducted to assess the overall quality of the included studies and to identify potential sources of bias. Two RCTs were clearly identified as prospective, but due to missing details on allocation concealment and intervention blinding, their risk of bias was rated as moderate using the Cochrane tool. Three retrospective studies were classified as retrospective diagnostic performance validation studies and were rated as moderate to high quality by the STARD guidelines, though they still carried a risk of bias due to limited model generalizability. Nine non-interventional observational studies employed standardized protocols and assessment systems, along with simulated real-world clinical scenarios, and were therefore categorized in this review as non-interventional structured simulated observational studies. Most were rated as high quality by the STROBE guidelines, although a frequent source of bias was reliance on expert-based subjective scoring. Two cross-sectional content analysis studies were evaluated as moderate quality by the DISCERN tool, with subjective scoring and corpus dependency potentially affecting the stability of LLM outcomes (Table 2).

**TABLE 2:** Summary of methodological quality and risk of bias in included studies.

References	Study type	Primary evaluation tool	Quality rating	CLEAR-LLM assessment	Limitations
Yang et al. (23)	Prospective randomized controlled trial	Cochrane RoB 2.0	Moderate quality (1 high-risk item)	Moderate risk	No blinding or allocation concealment Unclear model version Indirect outcomes Small and region-specific sample
Xie et al. (21)	Retrospective diagnostic performance validation	STARD	High quality (28/30)	Low to moderate risk	Subjective metric bias Single-center, medium-sized with no external validation
Du et al. (26)	Structured simulated observational study	STROBE	High quality (22/24)	Low risk	Lacked actual behavioral or long-term outcome measures Small sample limits statistical power
Temel et al. (14)	Retrospective diagnostic performance validation	STARD	Moderate quality (27/30)	Moderate risk	No context memory Lacks clinical or behavioral outcome Limited generalizability
Zhu et al. (15)	Retrospective diagnostic performance validation	STARD	High quality (28/30)	Moderate risk	Limited to image grading tasks Difficulties in identifying severe KOA Single-center
Pagano at al. (13)	Structured simulated observational study	STROBE	High quality (22/24)	Low risk	No adherence metrics Limited real-world complexity
Fahy et al. (20)	Cross-sectional content analysis	DISCERN	Moderate quality (45/80)	Moderate risk	Focused on a single surgical type Subjective scoring
Li et al. (29)	Structured simulated observational study	STROBE	High quality (22/24)	Low risk	Evaluation bias unclear Incomplete source referencing
You et al. (46)	Prospective randomized controlled trial	Cochrane RoB 2.0	Moderate quality (1 high-risk item)	Moderate risk	No blinding Simulation-based data
Cao et al. (16)	Structured simulated observational study	STROBE	High quality (22/24)	Low risk	Limited question scope Missing technical details of model Subjective scoring
Musbahi et al. (47)	Structured simulated observational study	STROBE	High quality (22/24)	Low risk	Indirect indicators LLM variability uncontrolled
Fahy et al. (31)	Cross-sectional content analysis	DISCERN	Moderate quality (62/80)	Moderate risk	LLM only evaluated at answer level Incomplete model info Limited question scope No blinding
Yang et al. (25)	Structured simulated observational study	STROBE	High quality (23/24)	Low to moderate risk	Subjective evaluation criteria Unclear question source Lacked patient perspective
Pagano et al. (24)	Retrospective simulated observational Study	STROBE	High quality (23/24)	Moderate risk	No blinding or replication No randomization or iterative testing
Kuroiwa et al. (22)	Structured simulated observational study	STROBE	High quality (22/24)	Moderate to high risk	Only simulation-based cases Missing technical details of model No formal rating scales Run frequency not reported
Gürses et al. (30)	Structured simulated observational study	STROBE	High quality (22/24)	Moderate risk	Moderate sample Lacks external validation

CLEAR-LLM, checklist for evaluating the methodological quality of LLM-based research; RoB, risk of bias tool; STARD, standards for reporting of diagnostic accuracy studies; STROBE, strengthening the reporting of observational studies in epidemiology; KOA, knee osteoarthritis; LLM, large language model.

**FIGURE 2:**
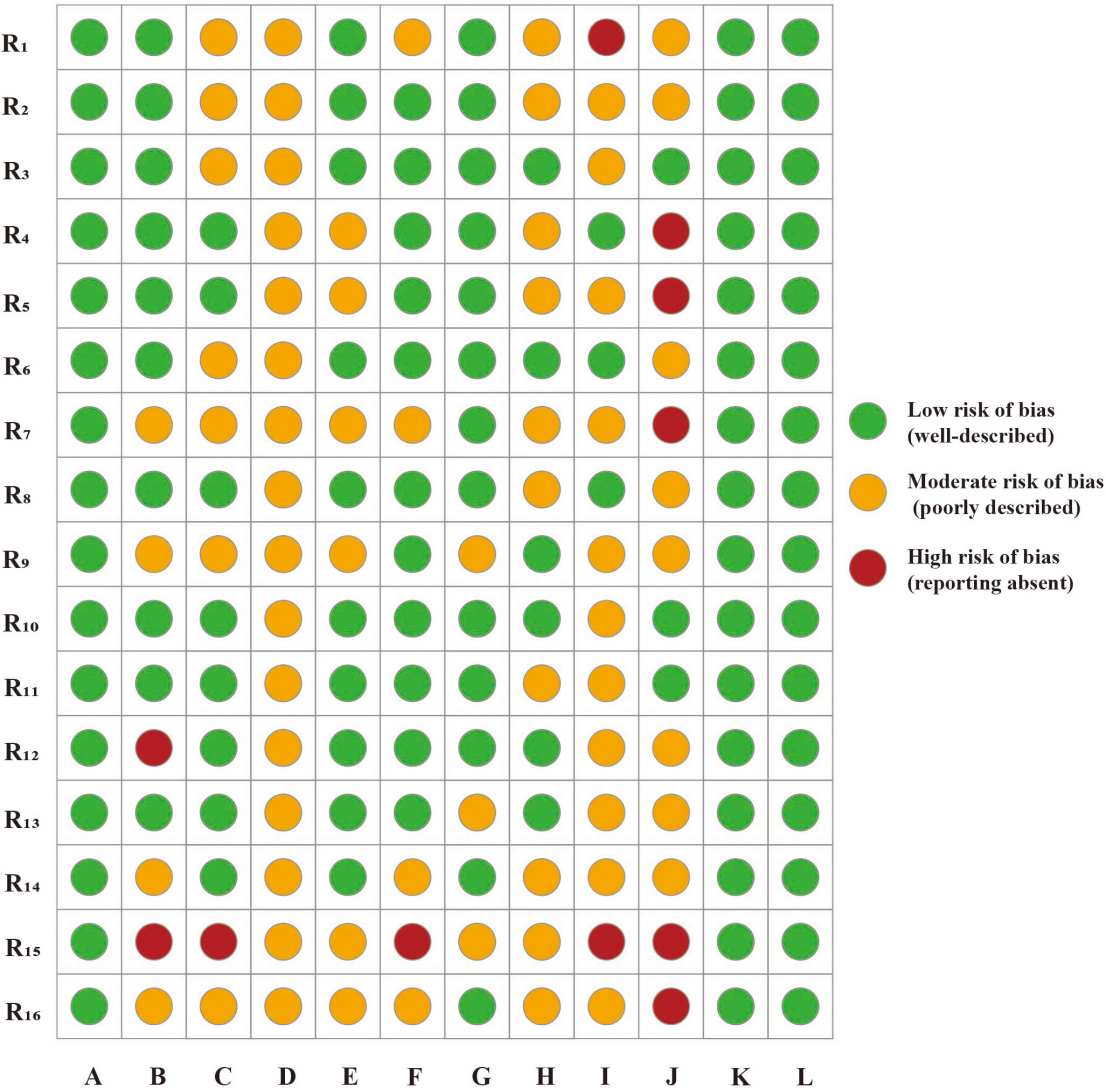
Risk assessment of CLEAR-LLM. R_1–16_, research 1–16; A, clarity of research objectives; B, control design; C, data sources and transparency; D, model description; E, prompt design; F, role of human evaluators; G, output evaluation and quantification; H, patient-relevance indicators; I, sample size and representativeness; J, bias control; K, ethical considerations; L, discussion of limitations.

Based on the CLEAR-LLM framework, the 16 included studies were further evaluated across multiple dimensions. A low risk of bias was observed in dimensions A (clarity of research objectives), K (ethical considerations), and L (discussion of limitations), demonstrating consistency in research aims, ethical compliance, and structural articulation in LLM-based KOA studies. In dimensions such as B (control design), E (prompt design), and G (output evaluation and quantification), most studies demonstrated a low risk of bias, well-defined experimental designs and robust evaluation strategies. In contrast, a moderate risk of bias was frequently observed in dimensions C (data sources and transparency), D (model description), F (role of human evaluators), and H (patient-relevance indicators), indicating limited reporting detail and a tendency toward incomplete or indirect evaluations. Dimensions I (sample size and representativeness) and J (bias control) demonstrated the highest bias levels, underscoring critical limitations in current LLM research on KOA, particularly insufficient sample design and inadequate bias control strategies (Figure 2).

### 3.3 Performance analysis of LLMs

**FIGURE 3:**
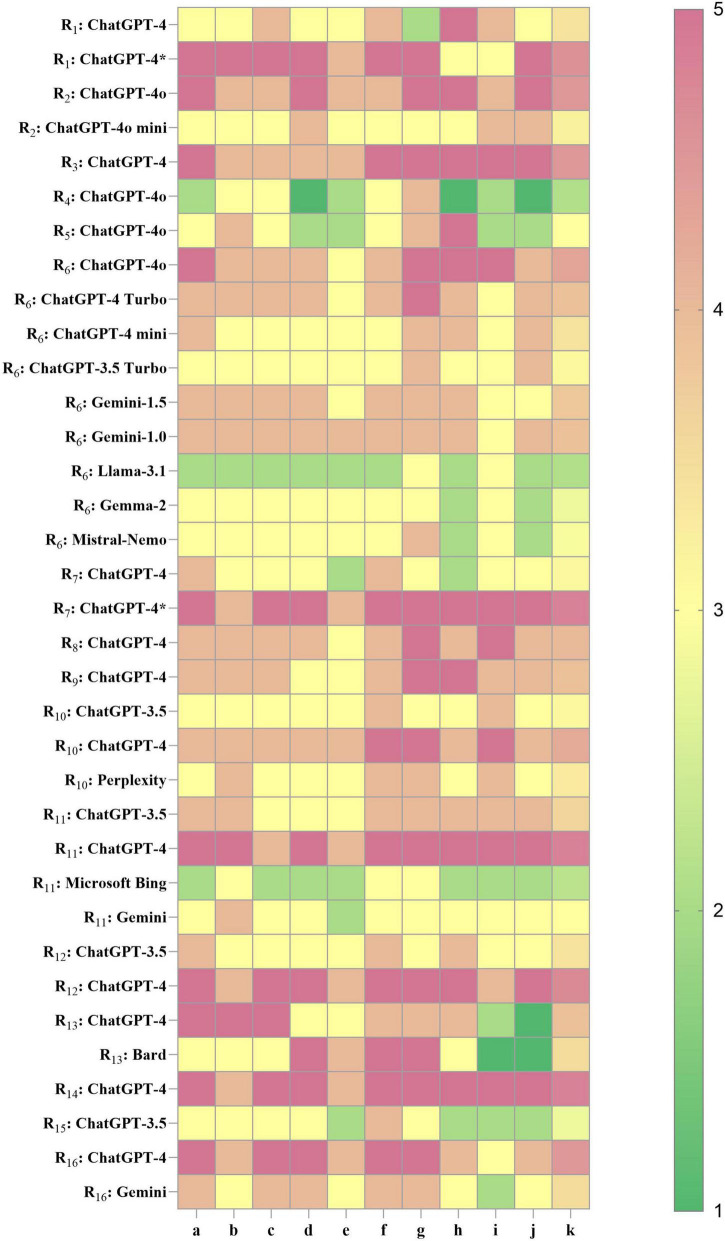
Performance heatmap of LLMs in KOA in current research. Higher values indicate better performance. R_1–16_, research 1–16; a, accuracy of medical content; b, contextual coherence; c, interpretability of medical terminology; d, clinical usefulness; e, hallucination control; f, safety and ethical compliance; g, structured output; h–j, flexible dimensions (detailed in Supplementary Materials); k, overall composite score. ChatGPT-4* , LLM fine-tuned based on KOA knowledge.

In the included studies, some cases involved multiple LLMs participating in KOA-related clinical tasks. Accordingly, based on the multidimensional CliMA-10 scoring system, performance analysis was conducted on 29 LLMs across 16 studies. The overall scores ranged from 2.1 to 4.8, exhibiting a pronounced right-skewed distribution, indicating that mainstream LLMs currently achieve an upper-moderate level of performance in KOA clinical tasks. Specifically, 5 LLMs achieved high performance (>4.5), 6 attained upper-moderate performance (4.0–4.5), 18 reached moderate performance (3.0–3.9), and 6 demonstrated relatively low performance (<3.0) (Figure 3).

Among the core evaluation dimensions, the average scores for dimension a (accuracy of medical content) and dimension c (interpretability of medical terminology) were consistently around 4, suggesting that most LLMs exhibit sufficient domain knowledge and professional communication capacity in the KOA domain. However, dimension g (structured output) showed substantial variability across models, which directly affected their adaptability to tasks such as clinical note summarization, disease staging, and structured history-taking. The ChatGPT-4 series demonstrated high adaptability across multiple studies and KOA-specific clinical applications. In particular, the fine-tuned variant, Knee-ChatGPT, achieved the highest scores (4 or 5) across key dimensions, demonstrating strong potential for deployment in real-world clinical practice. ChatGPT-4o and its Turbo variants exhibited strong generalizability, but showed some variability across different studies and tasks, suggesting that the models may be sensitive to input format, task type, and context settings. In contrast, ChatGPT-3.5 produced lower composite scores (2.8–3.6), suggesting that it may be more appropriate for educational, informational, or other low-risk applications. However, it is not advisable for use in high-precision or safety-critical clinical decision support systems. Non-OpenAI models, including Gemini (1.0/1.5/advanced), LLaMA 3.1, Gemma 2, and Mistral-Nemo, generally demonstrated moderate to low performance (2.8–3.9). Notably, these models showed suboptimal performance in dimension g (structured output) and dimension e (hallucination control), with LLaMA 3.1 and Gemma 2 scoring between 2 and 3 in the latter. This indicates an increased risk of factual inaccuracies in medical content generation, highlighting the necessity for further optimization prior to clinical deployment.

## 4 Discussion

### 4.1 Application tasks and model adaptability

LLMs have been increasingly investigated for clinical applications across various domains in orthopedics, including improving the readability of patient education materials for spinal disorders (10), and supporting medical knowledge popularization related to anterior cruciate ligament injuries (11). However, in the context of KOA, a prevalent chronic degenerative condition, the systematic clinical application of LLMs remains nascent, with limited comprehensive evaluations and few cross-model comparisons. This study presents the first systematic review of LLMs applications in KOA, synthesizing empirical evidence from the past decade. It provides a structured analytical framework and a tiered performance evaluation spanning application scenarios, task categories, model behaviors, and methodological rigor.

Clarifying task stratification helps delineate the boundaries of LLM applications in real-world healthcare and provides a reference framework for risk management in future research and clinical practice. Currently, LLM applications in KOA are primarily concentrated in three core domains: clinical text generation, imaging-assisted diagnosis, and patient education with health management support. Among these, medical text generation is particularly well suited to the capabilities of LLMs and is often adopted in early clinical deployments (12). For example, Stefano Pagano et al. demonstrated that, using only a structured patient questionnaire as input, an LLM could enable “contact free” preliminary KOA screening, achieving high performance in medical terminology interpretation and semantic coherence (13). In the field of imaging-assisted diagnosis, LLMs remain at an exploratory and validation stage. ChatGPT-4o shows relatively high accuracy and recognition capability in interpreting knee joint radiographs and providing preliminary KOA assessments. Nonetheless, for more granular grading tasks, there is still no established evaluation framework, and the model tends to display significantly low sensitivity and a marked underestimation of severity (14, 15). In the domain of patient education and health management, researchers have developed question–answer datasets covering a wide range of KOA-related issues, from etiology to treatment recommendations, based on real-world scenarios (16). From a clinical risk perspective, LLMs pose relatively low risk in patient education and health communication tasks, where the primary goals are to enhance patient understanding and improve adherence. Even if inaccuracies occur, they can be supplemented and corrected by healthcare professionals. In contrast, tasks such as imaging-based prescreening, clinical note summarization, and medical question answering involve moderate risk, requiring physician oversight to ensure accuracy and reliability of the interpretations. For high risk tasks, including diagnostic stratification, treatment planning, and personalized intervention recommendations, the outputs of LLMs may directly influence clinical decision-making and patient safety. Therefore, their use must be approached with caution, making them more suitable as reference or assistive tools rather than as independent decision-making agents.

Significant performance disparities were observed among different LLMs and task types. Structured output tasks (e.g., medical summaries, diagnostic grading) were more discriminative of model capabilities, whereas open-ended questions and patient education tasks emphasized fluency and general medical knowledge, where inter-model performance differences were comparatively minor. High-performing models, particularly the ChatGPT-4 series, demonstrated robust adaptability in core clinical tasks, with superior performance in structured text generation, medical terminology interpretation, and multi-turn dialogue when compared to ChatGPT-3.5 and non-OpenAI models. Gemini has approached ChatGPT in terms of natural language fluency and conversational experience; however, it still demonstrates limitations in the depth of medical knowledge coverage and safety control (17). Open-source models such as LLaMA offer clear advantages in flexibility and transparency (18), facilitating task customization and secondary development by researchers. Nonetheless, they remain relatively weak in hallucination control and stability in clinical tasks, and are prone to generating factually inconsistent responses in complex diagnostic scenarios (19). Fine-tuned models such as Knee ChatGPT exhibited the best task-specific performance, benefiting from prompt sensitivity and training on domain-specific KOA corpora (20). Some models exhibited significant fluctuations in output quality when prompts were not optimized, suggesting that current LLMs remain in a stage of high performance yet limited robustness. Future research should avoid bias arising from reliance on a single vendor’s model and instead conduct systematic comparisons across models from different providers to assess their adaptability and safety in clinical applications, thereby yielding more comprehensive and objective conclusions.

In summary, the adaptability of LLMs to KOA-related clinical tasks is influenced by multiple interrelated factors, including task complexity, input structure, and training methodology. Current applications remain predominantly superficial, involving clearly defined outputs, low clinical risk, and high controllability. In contrast, more complex tasks such as personalized treatment planning, disease progression prediction, and cross-modal diagnostic support remain in the exploratory stage due to limitations such as data accessibility, lack of standardized evaluation metrics, and insufficient model generalization capabilities.

### 4.2 Study quality and evaluation frameworks

#### 4.2.1 Methodological quality assessment and risk of bias

The evaluation of methodological quality aimed to identify potential sources of bias in the design, execution, and reporting of the studies. Depending on the study type, established tools including Cochrane RoB 2.0, STARD, STROBE, and DISCERN were used as primary frameworks for quality appraisal. Overall, most studies demonstrated well-organized reporting and clearly defined workflows, with the majority rated as moderate to high quality. However, several common sources of bias were identified during the review process. These included insufficient reporting of randomization and blinding procedures, small sample sizes with limited representativeness, reliance on subjective evaluation metrics lacking quantitative validation, and the absence of long-term follow-up data for evaluating clinical efficacy, with outcome measures often limited to indirect indicators.

Both prospective RCTs evaluated using the Cochrane RoB 2.0 tool were rated as having some concerns. Although their designs satisfied formal criteria for high-level clinical evidence, neither study reported allocation concealment or blinding procedures, raising concerns about selection and observer bias. Although their designs satisfied formal criteria for high-level clinical evidence, neither study reported allocation concealment or blinding procedures, raising concerns about selection and observer bias. Additionally, both studies did not specify the LLM version used, and primary outcomes were indirect measures (e.g., knowledge acquisition) rather than behavioral changes or long-term clinical benefits. Small sample sizes and limited geographic diversity further compromised external validity and hindered generalizability.

Three retrospective diagnostic performance studies were evaluated using the STARD criteria and were generally rated as high quality, especially regarding protocol standardization and clarity in reporting diagnostic metrics. However, key limitations included single-center data, absence of external validation datasets, and reliance on subjective outcome interpretation, potentially affecting real-world generalizability.

Nine simulation-based structured observational studies were assessed using the STROBE checklist. Although most scored above 22 points, indicating strong methodological rigor, critical limitations remained. These included reliance on simulated scenarios rather than real patient cases, use of outcome measures based on subjective scoring or question-answering accuracy, and inadequate sample sizes for statistical power. Several studies did not report whether evaluators were blinded or whether interrater reliability was assessed, increasing the risk of interpretive bias.

The two cross-sectional content analysis studies were evaluated using the DISCERN tool and were both rated as moderate quality. These studies focused on the qualitative and quantitative assessment of LLM-generated content, relying heavily on subjective scoring. However, their validity was limited by non-standardized rating criteria, corpus dependency, and potential evaluator bias. Furthermore, some studies did not report whether blinding was applied or how many times the models were run, which reduces reproducibility and undermines the reliability of the findings.

#### 4.2.2 LLM specific appraisal with the CLEAR-LLM framework

Given the distinctive characteristics of LLMs in the medical domain, the CLEAR-LLM tool was also employed as a supplementary quality assessment framework. This tool complements primary evaluation instruments by focusing on study structure, logical transparency, and potential bias control, thereby offering an integrated perspective on methodological strengths and limitations in terms of structural and logical completeness.

In dimension A (clarity of research objectives), all studies clearly articulated their aims in the introduction, addressing application areas such as patient education, KOA grading, and information quality evaluation. In dimension B (comparative design), most studies established structured comparison frameworks, such as comparisons between different LLM versions (e.g., ChatGPT-4o versus ChatGPT-4o mini) (21) or between human expert outputs and model-generated content (15). However, several studies lacked robust control groups or omitted human benchmarks, thereby weakening causal inference on model effectiveness. In dimension C (data sourcing and transparency), approximately half of the studies utilized real clinical data with clearly documented sources, preprocessing steps, exclusion criteria, and appended model outputs, thereby improving reproducibility (15). In contrast, a minority of studies did not disclose data sources, prompts, or responses, and in some cases relied solely on prototypical scenarios instead of actual patient data (22). In dimension D (model description), all studies explicitly identified the models used, primarily from the ChatGPT series, along with others such as Claude and Gemini. However, most lacked detailed documentation of model versions, API parameters (e.g., token limit), training context, and invocation methods, limiting reproducibility in future studies. dimension E (prompt design), most studies implemented standardized prompt formats, often incorporating both physician and patient perspectives (13). However, few studies investigated the influence of prompt variability on output quality, leaving an important methodological factor insufficiently addressed. In dimension F (the role of human evaluators), the evaluation process incorporates blinded scoring methods such as expert review mechanisms, clinician ratings, and patient preference assessments, with reports on evaluator composition, scoring procedures, and consistency assessments (14). Nonetheless, some failed to specify rater characteristics or include clinician input (23, 24), limiting interpretability due to the absence of cross-validation procedures. In dimension G (quantification of output quality), quantitative indicators such as accuracy, recall rate, and F1 score are clearly defined, and significance tests or cross-task comparisons are performed. Some studies combine binary classification or subjective evaluation, but lack detailed quantitative indicators (22, 25). In dimension H (patient-relevance indicators), some studies incorporate patient feedback or preferences into the primary outcome variables, focusing on the understandability, trust, and user experience of patients regarding suggestions made by LLM (26). While these outcomes improve clinical relevance, numerous studies rely solely on simulated content or have physicians make judgments instead of patients, which are indirect indicators and struggle to accurately represent the model’s true impact on changes in patient behavior or health outcomes, leading to insufficient clinical interpretability (21). In dimension I (sample size and representativeness), the distribution of research sample sizes varies considerably, with most studies relying on small samples and single-center data. Some studies have introduced multinational assessors or public datasets to enhance sample breadth and representativeness (13). However, overall, the explanations regarding sample source, stratification criteria, and coverage population remain insufficient, thereby limiting the external validity of the research. In dimension J (bias control), several studies attempted to mitigate selection bias by employing preregistered protocols, strict inclusion and exclusion criteria, multicenter data sources, or blinded scoring. However, the overall implementation quality was inconsistent. For example, a minority of studies explicitly reported participants’ backgrounds, case sources, and stratification criteria, which helped reduce the risk of selective bias. In contrast, many studies did not provide detailed information on sample acquisition processes and were largely based on single-center, small-sample designs, thereby limiting the representativeness and external generalizability of their findings. In dimension K (ethics), all studies have obtained ethical approval or declared no risk, with informed consent explanations provided for patient recruitment, thereby ensuring compliance with basic ethical standards. In dimension L (limitations discussion), the studies prudently address issues such as model version variability, insufficient sample sizes, poor cross-cultural adaptability, and the pronounced subjectivity of human scoring in their conclusions.

### 4.3 Performance evaluation

Previous AI studies related to KOA have primarily focused on traditional machine learning and computer vision models, especially in the context of imaging-based recognition and classification tasks (such as the application of convolutional neural network models in automated grading of X-rays and MRIs) (27). In comparison, LLMs with their core rooted in natural language processing, exhibit distinctive advantages in workflows within clinical practice that heavily depend on unstructured language information, such as medical history collection, question-and-answer interpretation, patient education, and health advice generation.

This study introduces the novel CliMA-10 multidimensional scoring framework, characterized by task-adaptive orientation and flexible dimension design. In image-assisted diagnosis tasks, emphasis is placed on structured output capabilities and medical content accuracy, while in patient education and personalized management tasks, greater importance is attached to the combination of term interpretability, ethical safety, and empathy. Unlike most previous studies that rely solely on single-dimensional metrics (such as accuracy rates, F1 scores, satisfaction ratings) (28), CliMA-10 supports the configuration of task types and weights prior to scoring, enabling fair comparisons across models and tasks, as well as clinical deployment evaluations. Overall scoring results reveal that mainstream LLMs have achieved a high level of performance in term interpretability, medical knowledge articulation, and linguistic coherence. Notably, the ChatGPT-4 series demonstrates exceptional performance in structured generation, offering clear, well-organized diagnostic recommendations and educational materials with broad adaptability. In contrast, non-OpenAI models like Gemma and LLaMA show relatively lower scores in structured expression capabilities and hallucination control. Consequently, scientific evaluation of the performance of LLMs in KOA clinical applications should be grounded in task-specific comparative analyses rather than generalized rankings.

In empirical studies, the performance of LLMs in KOA-related clinical tasks varied significantly and can be attributed to five key underlying mechanism. Firstly, the depth of embedding in medical corpora and the precision of knowledge coverage determine the boundaries of medical concept expression and inference. The accuracy of LLMs in KOA tasks and the quality of medical terminology used are directly influenced by the extent of coverage of orthopedic, radiological, and rehabilitation knowledge within their pre-trained datasets. For instance, ChatGPT-4 demonstrates remarkable proficiency in interpreting treatment guidelines, achieving an accuracy rate of 96.4% in tasks related to the American academy of orthopaedic surgeons (AAOS) and the Canadian orthopaedic association (COA) guidelines (29). It also provides detailed explanatory narratives according to recommendation grades. In contrast, models like Gemma and Bard frequently offer intervention suggestions inconsistent with guidelines when addressing issues related to hyaluronic acid injections, indicating limited coverage of medical standard guidelines within their training data, thereby impacting content accuracy and professionalism (25). Secondly, the ability to produce structured outputs and the strategy for semantic organization are crucial determinants of a model’s practicality in standardized tasks. The GPT-4o model exemplifies high utility by automatically converting free-text into the standardized WORMS scoring template, ensuring clear structure and concise key points, thus significantly reducing physicians’ reading time by 58.6% and enhancing accuracy to 96.2% (21). Additionally, GPT-4o can autonomously generate phased structured intervention pathways, with a match rate of up to 74% with physiotherapy plans. In comparison, the Gemini model frequently presents fragmented and disorganized outputs, hampering its practical application (30). Thirdly, the ability to control hallucinations is paramount to the credibility and safety of outputs. Some LLMs erroneously recommend treatments not supported by guidelines, such as early KOA treatment with hyaluronic acid injections, and may even propose invasive treatment options without proper disease classification input. In contrast, ChatGPT-4 exhibits a lower hallucination rate and stronger task limited awareness across numerous tasks, often appending advisories to consult a physician, thereby bolstering the safety and trustworthiness of its outputs (31). Fourthly, the adaptability of input formats and semantic structures varies. In scenarios involving patient self-service or casual question and answer (Q&A), inputs are often non-standardized. The ChatGPT-4 series excels in the pragmatic generalization of natural language, adeptly parsing descriptions like “my knee feels stuck” and accurately identifying them as “joint dysfunction.” By contrast, models such as GPT-3.5 and Perplexity demonstrate higher misjudgment rates in handling such colloquial expressions. By adjusting the input format, such as removing the “My knee” prefix and enhancing the directive with “please list possibilities,” GPT-3.5’s accuracy can improve from 64% to a range of 88%–92% (22). Fifthly, whether a model has undergone fine-tuning in the medical domain significantly affects its performance. In the educational tasks regarding high tibial osteotomy, the “Knee Guide,” refined using iterative bootstrapped fine-tuning and reinforcement learning with human feedback methods, outperforms the original GPT-4 in both readability and content completeness (flesch–kincaid grade level scores of 8.2 versus 13.9, and DISCERN scores of 45.9 versus 38.4), while also reducing the textual difficulty to a recommended readability level (20).

### 4.4 Limitations

This study did not perform a meta-analysis, primarily for the following reasons. First, there was substantial heterogeneity among the included studies in terms of model types, task settings, and evaluation metrics, which precluded the formation of a comparable quantitative data structure. Second, the majority of the studies were exploratory in nature, and their outcomes were predominantly assessed using Likert scales or subjective ratings, making it difficult to extract standardized effect sizes. Third, several studies did not report essential statistical parameters such as standard deviations or sample sizes, further limiting the feasibility of data integration. Therefore, this study presents the main findings using a narrative synthesis approach and combines them CliMA-10 for hierarchical categorization to maintain the interpretability and methodological rigor of the analysis.

Nevertheless, several important limitations should be acknowledged. First, the majority of included studies were observational in nature. Although some simulation experiments are designed with rigor, the number of high-quality RCTs remains limited, with only two studies possessing prospective interventional characteristics, resulting in an overall evidence level that tends to be moderate to low. Moreover, the majority of studies focused primarily on immediate outputs, while lacking systematic evaluation of long-term clinical outcomes in KOA patients, such as improved adherence, functional recovery, and quality of life. The absence of follow-up and longitudinal observation further constrained the strength of external validation. Second, many performance metrics relied on expert subjective ratings and Likert scales. Although some studies incorporated blinded assessment or repeated ratings, subjectivity in the evaluation process could not be fully eliminated. The lack of objective indicators and multimodal evidence increases the risk of observer bias and limits comparability across studies. Thirdly, no widely validated tool for evaluating LLMs in medical contexts currently exists. Although this review applied CLEAR-LLM and CliMA-10 as auxiliary frameworks for quality and performance appraisal, both remain at an early stage, lacking cross-institutional and cross-platform validation, and thus requiring further establishment of external reliability. Fourth, most existing studies drew upon standardized cases, public imaging datasets, or simulated Q&A tasks, with limited integration of real-world clinical data. The paucity of multicenter, large-sample studies involving real patient participation restricts both generalizability and ecological validity. In addition, the inherent stochasticity of LLM outputs remains an overlooked issue (32). Most studies analyzed only single-run results, without recording or controlling for variability across multiple runs. This limitation extends beyond the present review to the broader body of LLM research. Particularly in clinical decision-making tasks, such variability not only reduces reproducibility but may also undermine the reliability of conclusions.

From a technological application perspective, LLMs still face significant challenges in clinical deployment for KOA and similar diseases. Notably, the generation of hallucinated content remains a persistent issue. Even high-performance models such as ChatGPT-4o may produce factually inaccurate outputs in scenarios involving diagnostic reasoning or treatment recommendations (33). Furthermore, institutional constraints related to patient data privacy, ethical compliance, and information security restrict LLMs from accessing real-world multimodal data, including medical records, imaging, and lab results hindering their embedded deployment and affecting their safety and clinical usability (34). In addition, the deployment of advanced models typically requires high computational resources and stable internet connectivity, conditions not readily available in primary care settings or developing countries. Limited technical literacy among healthcare providers also impedes broader implementation of LLMs across diverse healthcare environments (35). Finally, there is currently no mature ethical governance framework to standardize issues related to human–AI collaboration, hallucination control, and the traceability of medical decision-making (36). While several scholars have proposed operational standards for medical LLMs, formal guidelines have yet to be established (37).

### 4.5 Future research directions

#### 4.5.1 Dialogue-based clinical management centered on KOA

The current KOA clinical pathway emphasizes continuous monitoring of symptom progression, imaging changes, and functional deterioration. Patients typically undergo a diagnostic process involving “vague symptom complaints–functional assessment scale completion–imaging-assisted diagnosis–intervention recommendation feedback” (38). LLM can be used to design staged dialogue templates based on this process, transforming the clinical pathway into an interactive decision-making workflow. At present, most LLMs are limited to single-turn question answering or static information generation. Future research should focus on developing dialogue-based diagnostic and therapeutic systems, enabling LLMs to participate in or simulate the full continuum of the patient journey from early complaints of pain to mobility restrictions and treatment response. These systems should support context-aware, continuous reasoning across multiple dialogue rounds (39). For instance, adaptive questioning pathways could be created to dynamically adjust the dialogue flow based on user input, detect vague or incomplete patient descriptions, and trigger targeted follow-up questions. If a patient indicates prior exposure to a specific treatment, the system could bypass first-line recommendations and instead initiate relapse management or alternative therapy pathways.

#### 4.5.2 Multi-task prompt template library for KOA applications

Currently, prompts are mostly manually written and debugged, which is costly. In KOA applications, image-assisted tasks require accurate information extraction and structured scoring, while clinical decision-making tasks emphasize logical consistency, semantic interpretation, and personalized recommendations (40). In future research, researchers will develop a multi-task prompt template library tailored for KOA scenarios and design a visual prompt control interface, enabling medical staff to quickly select or customize prompt structures based on task objectives, patient characteristics, and input formats. When patient information is entered, the system can automatically recommend prompt templates featuring “risk assessment modules” and “non-pharmacological intervention priority,” enhancing the specificity and safety of LLM outputs.

#### 4.5.3 Personalized management of KOA in real-world settings

At present, most LLMs generate recommendations are based on idealized clinical standards, with limited sensitivity to real-world variables such as regional medical resource availability, economic constraints, and linguistic diversity (41). Future research should focus on enhancing the adaptability and practical value of LLMs in real-world KOA management scenarios. For example, incorporating health economic data (common treatment costs, medical insurance reimbursement scope) to estimate patients’ affordability can provide more personalized (42), low-cost intervention recommendations, thereby enhancing social equity. Additionally, in real-world scenarios, global patients rarely use standard language to describe KOA symptoms. Future research should enhance the model’s multilingual parsing capabilities to align with users’ linguistic and cultural backgrounds.

#### 4.5.4 Trustworthy lLM-based clinical decision-making in KOA

In KOA clinical management, the trust of physicians and patients in AI-generated recommendations depends largely on whether the AI can explain its conclusions (43). Recent studies indicate that LLMs often perform poorly in ensuring the authenticity of information sources when generating medical answers (44). Future research should therefore explore evidence-tracing mechanisms, whereby diagnostic or therapeutic recommendations are accompanied by explicit source annotations or references. From the perspective of output consistency, LLMs are subject to inherent randomness, leading to variations in results given identical inputs. Subsequent studies could incorporate repeated runs and report test–retest reliability, such as Cohen’s kappa for categorical outcomes or Bland–Altman analysis for continuous variables (45), while standardizing the reporting of model versions, parameter settings, and random seeds. For typical KOA-related tasks (e.g., Kellgren-Lawrence grading, risk factor identification, and treatment recommendation), stability thresholds and non-inferiority margins can be predefined. By structuring key information into analyzable fields, agreement rates and semantic similarity could then be calculated, providing a quantitative framework for assessing the stability of model outputs.

## 5 Conclusion and future perspectives

LLMs demonstrate robust capabilities in medical language comprehension and task adaptability, making them suitable for diverse clinical tasks within the domain of KOA. At present, the field remains constrained by limited sample representativeness, substantial methodological heterogeneity, and an unclear path for clinical validation. Future research should rely on real-world clinical data to drive model optimization, establish a unified framework for quality assessment, and strengthen cross-model comparative analyses, thereby reducing overreliance on any single vendor. Furthermore, systematic support at ethical, technical, and regulatory levels will be essential. Only by overcoming these methodological limitations can LLMs provide reliable support for the clinical management of KOA.

In summary, LLMs hold considerable promise for the management of KOA and other orthopedic disorders. This review provides a comprehensive overview of the current research landscape and methodological quality, identifies key developmental bottlenecks and directions for optimization, and underscores the potential value of LLMs in supporting diagnosis, treatment, and health management. Nevertheless, the findings should be interpreted with caution, as the present evidence represents exploratory insights rather than mature clinical proof.

## Data Availability

The original contributions presented in this study are included in this article/Supplementary material, further inquiries can be directed to the corresponding authors.

## Footnotes

1
https://doi.org/10.17605/OSF.IO/479R8
