# Supplementary material for: Clinical applications of large language models in knee osteoarthritis: a systematic review
Source: Front Med (Lausanne). 2025 Nov 19;12:1670824. doi: 10.3389/fmed.2025.1670824 (PMC12672416; doi:10.3389/fmed.2025.1670824)
Supplement: Supplementary file 1 [file Supplementary_file_1.docx]

Supplementary Material

# Search strategies for various databases

**PubMed**

("large language model"[Title/Abstract] OR "large language models"[Title/Abstract] OR "LLM"[Title/Abstract] OR "LLMs"[Title/Abstract] OR "ChatGPT"[Title/Abstract] OR "GPT"[Title/Abstract] OR "GPT-2"[Title/Abstract] OR "GPT-3"[Title/Abstract] OR "GPT-4"[Title/Abstract] OR "GPT-5"[Title/Abstract] OR "BERT"[Title/Abstract] OR "BioBERT"[Title/Abstract] OR "PubMedBERT"[Title/Abstract] OR "RoBERTa"[Title/Abstract] OR "T5"[Title/Abstract] OR "Flan-T5"[Title/Abstract] OR "LLaMA"[Title/Abstract] OR "PaLM"[Title/Abstract] OR "Claude"[Title/Abstract] OR "Gemini"[Title/Abstract])

AND

("osteoarthritis"[MeSH Terms] OR "osteoarthritis"[Title/Abstract] OR "osteoarthritic"[Title/Abstract] OR "degenerative joint disease"[Title/Abstract] OR "articular cartilage degeneration"[Title/Abstract] OR "joint degeneration"[Title/Abstract] OR "knee osteoarthritis"[Title/Abstract] OR "hip osteoarthritis"[Title/Abstract] OR "knee OA"[Title/Abstract] OR "hip OA"[Title/Abstract] OR "osteoarthritis of the knee"[Title/Abstract] OR "osteoarthritis of the hip"[Title/Abstract])

**Embase**

('large language model':ti,ab OR 'large language models':ti,ab OR 'llm':ti,ab OR 'llms':ti,ab OR 'chatgpt':ti,ab OR 'gpt':ti,ab OR 'gpt-2':ti,ab OR 'gpt-3':ti,ab OR 'gpt-4':ti,ab OR 'gpt-5':ti,ab OR 'bert':ti,ab OR 'biobert':ti,ab OR 'pubmedbert':ti,ab OR 'roberta':ti,ab OR 't5':ti,ab OR 'flan-t5':ti,ab OR 'llama':ti,ab OR 'palm':ti,ab OR 'claude':ti,ab OR 'gemini':ti,ab) AND ('osteoarthritis':ti,ab OR 'osteoarthritic':ti,ab OR 'degenerative joint disease':ti,ab OR 'articular cartilage degeneration':ti,ab OR 'joint degeneration':ti,ab OR 'knee osteoarthritis':ti,ab OR 'hip osteoarthritis':ti,ab OR 'knee oa':ti,ab OR 'hip oa':ti,ab OR 'osteoarthritis of the knee':ti,ab OR 'osteoarthritis of the hip':ti,ab)

**Cochrane Library**

("large language model*" OR "large language models" OR LLM* OR LLMs OR ChatGPT OR GPT OR "artificial intelligence" OR AI)

AND

("osteoarthritis" OR "joint degeneration" OR "degenerative joint disease" OR "articular cartilage degeneration" OR "osteoarthritic")

**Web of science**

TS=("large language model*" OR "large language models" OR "LLM" OR "LLMs" OR "ChatGPT" OR "GPT" OR "GPT-2" OR "GPT-3" OR "GPT-4" OR "GPT-5" OR "BERT" OR "BioBERT" OR "PubMedBERT" OR "RoBERTa" OR "T5" OR "Flan-T5" OR "LLaMA" OR "PaLM" OR "Claude" OR "Gemini" OR "transformer model*" OR "pretrained language model*")

AND

TS=("osteoarthritis" OR "osteoarthritic" OR "degenerative joint disease" OR "articular cartilage degeneration" OR "joint degeneration" OR "knee osteoarthritis" OR "hip osteoarthritis" OR "knee OA" OR "hip OA" OR "osteoarthritis of the knee" OR "osteoarthritis of the hip" OR "chronic knee pain" OR "knee joint arthritis" OR "knee cartilage degeneration" OR "joint pain" OR "osteoarthritic knee" OR "degenerative arthritis" OR "cartilage degeneration" OR "inflammatory osteoarthritis" OR "knee joint degeneration")

# Dimensions and reference frameworks for the CLEAR-LLM quality assessment tool

| **Design dimension** | **Referenced frameworks** | **Description** |
| --- | --- | --- |
| 1. Clarity of research question | CONSORT-AI | Emphasizes the clarity of study objectives and the defined role of AI in the research. |
| 1. Control design | CONSORT-AI, ROBINS-I | Focuses on the presence of control groups or comparative interventions involving AI. |
| 1. Data source and transparency | TRIPOD-AI, DECIDE-AI | Evaluates dataset availability, openness, and reproducibility. |
| 1. Model description | MINIMAR, TRIPOD-AI | Requires disclosure of model architecture, training methods, and hyperparameters. |
| 1. Prompt design | No direct reference available | A novel LLM-specific dimension; original indicators are proposed. |
| 1. Role of human evaluators | DECIDE-AI | Clarifies human involvement in feedback, annotation, or evaluation processes |
| 1. Output evaluation and quantification | CLEAR | Assesses whether objective scoring metrics are used to evaluate LLM outputs. |
| 1. Patient-relevance indicators | CONSORT-AI, TRIPOD-AI | Determines whether outputs can be translated into meaningful patient-level insights or advice. |
| 1. Sample size and representativeness | TRIPOD, ROBINS-I | Evaluates sample adequacy and population generalizability. |
| 1. Bias control | ROBINS-I | Focuses on identifying and controlling potential sources of bias. |
| 1. Ethical considerations | CONSORT-AI | Assesses ethical approval, data privacy, and informed consent processes. |
| 1. Discussion of limitations | All frameworks | Reporting study limitations is a fundamental requirement across all major guidelines. |
| CONSORT-AI (<https://doi.org/10.1038/s41591-020-1034-x>)  TRIPOD-AI (<https://doi.org/10.1136/bmj.q824>)  ROBINS-I (<https://doi.org/10.1136/bmj.i4919>)  DECIDE-AI (<https://doi.org/10.1038/s41591-021-01229-5>)  MINIMAR (<https://doi.org/10.1093/jamia/ocaa088>)  CLEAR (<https://doi.org/10.48550/arXiv.2403.12776>) | | |

# Clinical language model assessment framework (CliMA-10)

CliMA-10 is designed to provide a multidimensional evaluation of large language models (LLMs) in clinical scenarios, focusing on accuracy, usability, safety, and communication clarity. Below are the definitions and scoring standards for the 7 core dimensions and 3 flexible dimensions

| **Core dimension (7 total)** | **Definition** | **Likert scoring criteria** |
| --- | --- | --- |
| 1. Accuracy of medical content | Whether the LLM output is based on accurate medical knowledge and free of misleading statements. | 1: Contains serious medical errors; potentially harmful or misleading.  2: Multiple factual inaccuracies or vague statements affecting credibility.  3: Minor inaccuracies or imprecise language; generally acceptable framework.  4: Accurate content with only slight ambiguity or weak phrasing.  5: Fully accurate, aligned with authoritative medical knowledge; professionally expressed. |
| 1. Contextual coherence | Whether the generated content is logically consistent, semantically coherent, and contextually aligned throughout. | 1: Severely fragmented; disjointed and hard to follow.  2: Frequent logical jumps and unclear paragraph flow.  3: Generally coherent with some unnatural transitions.  4: Mostly coherent with minor inconsistencies.  5: Fully logical and fluent; excellent contextual flow. |
| 1. Interpretability of medical terminology | Whether the model explains medical terms or abbreviations sufficiently for non-specialist readers. | 1: Heavy use of jargon with little to no explanation. 2: Inadequate clarification of key terms. 3: Some terms explained, partially helpful. 4: Most key terms are reasonably explained. 5: All key terms are clearly and accurately interpreted. |
| 1. Clinical usefulness | Whether the output provides actionable clinical insights, such as supporting decisions or identifying risks. | 1: No value or offers misleading suggestions. 2: Generic content lacking clinical relevance. 3: Somewhat informative but vague or incomplete. 4: Clear suggestions or structured content with practical value. 5: Highly specific and actionable, useful for clinical decision-making. |
| 1. Hallucination control | Whether the model avoids fabricating facts, citations, or unmentioned inferences. | 1: Obvious hallucinations that compromise trust.  2: Multiple hallucinated elements.  3: Minor or isolated hallucinations not affecting the core message.  4: Virtually hallucination-free and reliable.  5: Fully accurate and fact-based output. |
| 1. Safety and ethical compliance | Whether the content adheres to medical ethics and patient safety standards, avoiding overly aggressive or inappropriate advice. | 1: Contains unsafe or unethical recommendations. 2: Vague or potentially inappropriate content. 3: Generally safe with minor wording issues. 4: Ethically sound and clinically safe. 5: Strong emphasis on ethics and safety, with cautious and compliant language. |
| 1. Structured output | Whether the model provides well-organized output using tables, lists, or bullet points to enhance readability and information extraction. | 1: Disorganized content; hard to extract key points. 2: Poor structure with minimal formatting. 3: Some signs of structure but not well developed. 4: Clear formatting and visible organization. 5: Highly structured and easy to read with clear logical flow. |

| **Study** | **Flexible dimensions (based on actual application tasks)** (**3 total)** |
| --- | --- |
| Yang Y et al. | 1. User engagement; 2. Patient self-learning capability; 3. Potential to reduce physician workload |
| Xie et al. | 1. User preference; 2. Information extraction efficiency; 3. Contribution to reducing clinical workload |
| Du et al. | 1. Content generation efficiency; 2. Degree of personalization; 3. Patient readability |
| Temel et al. | 1. Image interpretation ability; 2. Inter-model consistency; 3. Deployability |
| Zhu et al. | 1. Image recognition capability; 2. Grading accuracy; 3. Inter-model consistency |
| Pagano at al. 2024 | 1. Response completeness; 2. Inter-model consistency; 3. Diagnostic sensitivity |
| Fahy et al. | 1. Patient readability; 2. Response quality; 3. Applicability of patient education materials |
| Li et al. | 1. Consistency of treatment suggestions; 2. Case analysis capability; 3. Clinical decision support capability |
| You et al. | 1. Adaptability of personalized treatment plans; 2. Fact-based feedback and monitoring; 3. Patient engagement |
| Cao et al. | 1. Accuracy; 2. Completeness of response; 3. Relevance to treatment and prevention |
| Musbahi et al. | 1. Decision-making consistency; 2. Confidence in decisions; 3. Applicability to treatment and prevention |
| Fahy et al. | 1. Accuracy of treatment plans; 2. Patient readability; 3. Traceability of cited literature |
| Yang J et al. | 1. Guideline concordance comparison; 2. Differentiation in recommendation identification; 3. Traceability of cited literature |
| Pagano et al. 2023 | 1. Consistency of treatment recommendations; 2. Diagnostic accuracy; 3. Clinical decision support capability |
| Kuroiwa et al. | 1. Cross-disease recognition consistency; 2. Reliability of self-diagnosis; 3. Clarity in referral recommendations |
| Gürses et al. | 1. Rehabilitation process logic adaptability; 2. Stability in non-English applications; 3. Support for individualized KOA rehabilitation |

**Weighted Composite Score Calculation Method:**

The total weight of core dimensions is 60%, and the total weight of flexible dimensions is 40%. Within each category, equal weighting is applied. That is, each core dimension carries a weight of approximately 8.57% (60% ÷ 7), and each flexible dimension carries a weight of approximately 13.33% (40% ÷ 3). The overall composite score S is calculated using the following formula:

$$S=\frac{1}{7}\cdot60\%\cdot\sum_{i=1}^{7} C_{i}+\frac{1}{3}\cdot40\%\cdot\sum_{j=1}^{3} S_{j}$$

Where C_i_ is the score of the *i*-th core dimension, and S_j_ is the score of the *j*-th flexible dimension.

**References:**

[1] Singhal K., Azizi S., Tu T., Mahdavi S.S., Wei J., Chung H.W., et al. Large language models encode clinical knowledge. Nature. (2023)620:172–180. doi: 10.1038/s41586-023-06291-2.

[2] Singhal K., Tu T., Gottweis J., Sayres R., Wulczyn E., Hou L., et al. Towards Expert-level Medical Question Answering with Large Language Models. arXiv. (2023) doi: 10.48550/arXiv.2305.09617. Preprint at.

[3] Liévin, V., Hother, C. E., Motzfeldt, A. G., & Winther, O. Can large language models reason about medical questions?. Patterns (New York, N.Y.). (2024)*5*(3), 100943. https://doi.org/10.1016/j.patter.2024.100943

[4] Gallifant, J., Afshar, M., Ameen, S., Aphinyanaphongs, Y., Chen, S., Cacciamani, G., et al. The TRIPOD-LLM reporting guideline for studies using large language models. *Nat Med*. (2025)31(1), 60–69. https://doi.org/10.1038/s41591-024-03425-5
